# Sparse identification of Lagrangian for nonlinear dynamical systems via proximal gradient method

**DOI:** 10.1038/s41598-023-34931-0

**Published:** 2023-05-16

**Authors:** Adam Purnomo, Mitsuhiro Hayashibe

**Affiliations:** Department of Robotics, Graduate School of Engineering, Tohoku University, Sendai, 980-8579 Japan

**Keywords:** Mechanical engineering, Applied mathematics, Computational science

## Abstract

The autonomous distillation of physical laws only from data is of great interest in many scientific fields. Data-driven modeling frameworks that adopt sparse regression techniques, such as sparse identification of nonlinear dynamics (SINDy) and its modifications, are developed to resolve difficulties in extracting underlying dynamics from experimental data. However, SINDy faces certain difficulties when the dynamics contain rational functions. The Lagrangian is substantially more concise than the actual equations of motion, especially for complex systems, and it does not usually contain rational functions for mechanical systems. Few proposed methods proposed to date, such as Lagrangian-SINDy we have proposed recently, can extract the true form of the Lagrangian of dynamical systems from data; however, these methods are easily affected by noise as a fact. In this study, we developed an extended version of Lagrangian-SINDy (xL-SINDy) to obtain the Lagrangian of dynamical systems from noisy measurement data. We incorporated the concept of SINDy and used the proximal gradient method to obtain sparse Lagrangian expressions. Further, we demonstrated the effectiveness of xL-SINDy against different noise levels using four mechanical systems. In addition, we compared its performance with SINDy-PI (parallel, implicit) which is a latest robust variant of SINDy that can handle implicit dynamics and rational nonlinearities. The experimental results reveal that xL-SINDy is much more robust than the existing methods for extracting the governing equations of nonlinear mechanical systems from data with noise. We believe this contribution is significant toward noise-tolerant computational method for explicit dynamics law extraction from data.

## Introduction

Researchers have attempted to develop models that can capture real-world phenomena since the early modern history of humanity. Such models are desirable because they can be used to devise solutions to real-world problems. The process of refining the hypotheses, which should be falsifiable so that it can be meaningfully tested^1^, from experimental data have been performed manually over the centuries; automation of these processes have long been of great interest to the scientific community.

Many attempts have been made to autonomously extract physical laws from data. With the abundance of data and inexpensive yet powerful hardware, deep learning-based methods have attracted considerable attention. The revolution of deep learning begins when LeCunn et al.^2^ and Krizhevsky et al.^3^ generalized the backpropagation algorithm for training multilayers networks. Deep learning has become popular in the robotics field due to its efficacy in solving complex robotic tasks and has been used extensively to model and control dynamical systems in recent years^4–6^. Moreover, deep learning models can approximate physical quantities such as the Hamiltonian^7^ and Lagrangian^8^ or mathematical quantities such as Koppman Eigenfunctions^9–11^ from dynamical systems. Koopman Eigenfunction is especially useful for nonlinear dynamical systems as it provides a framework to embed nonlinear dynamical systems into linear systems in an infinite Hilbert space^12,13^.

Another variant of neural networks, graph neural network, has also been used to obtain the dynamics model of interacting particle^14^. In this method, a deep learning model with a separable internal structure that provides an inductive bias was trained in a supervised manner. Xie et al. combine graph neural network and Koopman analysis to model the behavior of molecular dynamics in an unsupervised manner^15^. One caveat is that, deep learning models act as black boxes; they do not provide insight into how the observational variables affect and relate to each other.

In contrast to neural network-based methods, recent trends favor parsimonious white-box modeling with the lowest complexity to describe experimental data. Ground breaking work by Schmidt and Lipson^16^ demonstrated that it is possible to extract the governing mathematical expressions from observational data. Although symbolic regression can be used to determine the nonlinear differential equations that describe the system behavior, this approach tends to be expensive. Sparse identification of nonlinear dynamics (SINDy)^17^ models the nonlinear differential equations of dynamics as a linear combination of nonlinear candidate functions and obtains a parsimonious model through sparse regression^18,19^.

Although SINDy offers many applications across different fields^20–23^, it faces certain difficulties when the dynamics contain rational functions. The inclusion of rational functions in the library of candidate functions increases the size of the library significantly, making the sparse regression challenging. A modification of SINDy known as implicit-SINDy^24^, reformulates the SINDy problem into an implicit form to address this challenge; however this method is sensitive to noise due to non-convex optimization procedure^25^. SINDy-PI was proposed to improve the performance of implicit-SINDy, in terms of noise robustness by reformulating the problem into a convex optimization problem.

Although SINDy-PI is substantially more robust than implicit-SINDy, it can only obtain the correct dynamical structure with a noise magnitude on a scale of up to $10^{-3}$, which may not be sufficient for real-world applications. Furthermore, the predicted system may blow up when the denominator is equal to zero if an incorrect combination of denominator terms is discovered. Another SINDy variant that can identify rational functions is RK4-SINDy^26^ where it combines Runge-Kutta method with sparse identification method. Instead of reformulating the equation into implicit form, RK4-SINDy defines linear combination of nominators and denominators separately before combining the results. However, this method has only been tested on a rather simple rational equation. The possibility that incorrect denominators is found in more complex systems and may cause prediction that blows up still exists.

The principle of least action is fundamental to many dynamical systems^27^. This principle states that the trajectory that is selected by the system minimizes a certain cost function. This cost function is the so-called action, which is defined as the integral of the Lagrangian for an input evolution over a certain period. The Lagrangian has a desirable property compared to the underlying differential equations in that it is a single scalar quantity that contains all information predicting the system behavior. In robotics, the derivation of the dynamics is often initiated with the Lagrangian of the systems.

Several techniques have been proposed to approximate the Lagrangian from data using polynomial basis functions^28,29^. However, the approximation of the Lagrangian with polynomial basis functions is only useful for a particular trajectory of the system and is not likely to be generalized well across different initial conditions effectively. Lagrangian-SINDy^30^ is a SINDy-based method that is designed to extract the Lagrangian of the nonlinear dynamics and can retrieve the true form of the Lagrangian of several dynamical systems. Lagrangian-SINDy first builds an expression of total energy, which turns out be a Koopman eigenfunction with eigenvalue $\lambda =0$^31^, and adopts sparse regression by comparing the time derivative of the total energy to the net power that comes into the system.

However, the above paper noted that Lagrangian-SINDy is certain sensitive to noise and cannot recover the Lagrangian when the training data are corrupted by Gaussian noise even with a magnitude on the scale of $10^{-7}$. It was the remained challenge for this method. Because noise is always present in real-world systems, the development a method that is robust against noise is important for real-world applications.

In this study, we propose a method known as extended Lagrangian-SINDy (xL-SINDy), which can discover the true form of the Lagrangian and is more robust in the presence of noise than Lagrangian-SINDy and SINDy-PI. Unlike Lagrangian-SINDy which discovers Lagrangian through Hamiltonian, xL-SINDy directly finds the Lagrangian which will be explained in the problem formulation section. We demonstrate the effectiveness of xL-SINDy against different noise levels and compare its robustness with that of SINDy-PI in physical simulations using four dynamical systems: a single pendulum, cart-pendulum, double pendulum, and spherical pendulum.

**Figure 1:**
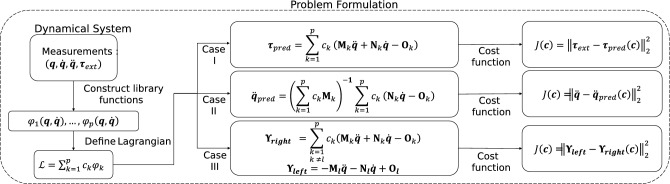
Block diagram of the proposed method (xL-SINDy). Depending on the case of the problem, a different cost function is constructed. Once the cost function is defined, the cost function is minimized by using the proximal gradient descent method.

## Results

### New problem formulation for Lagrangian acquisition

We consider a Lagrangian expression in the structure of a linear combination of nonlinear candidate functions. Let ${\varvec{q}} = (q_1, q_2, ..., q_n)$ be the configuration of a system in generalized coordinates. The Lagrangian of the system is expressed as

1 $$\begin{aligned}{}&\mathcal {L} = \sum _{k=1}^{p} c_k\phi _{k}({\varvec{q}},\dot{{\varvec{q}}}), \end{aligned}$$

where $\phi _{k}({\varvec{q}},\dot{{\varvec{q}}})$, $k=1,...,p$ is a set of nonlinear candidate functions and $c_{k}$, $k=1,...,p$ are the corresponding coefficients. We are interested in determining the value of ${\varvec{c}} = (c_1, c_2,..., c_p)$, in which we believe that most coefficients are zero. The Lagrangian of the system satisfies the Euler–Lagrange equations given by

2 $$\begin{aligned}{}&{\varvec{\tau }}_{ext} = \frac{d}{dt} \nabla _{\dot{{\varvec{q}}}}\mathcal {L} - \nabla _{{\varvec{q}}}\mathcal {L}, \end{aligned}$$

where $\left( \nabla _{{\varvec{q}}}\right) _i \equiv \frac{\partial }{\partial q_i}$. We consider three different scenarios: active systems where the external input ${\varvec{\tau }}_{ext}$ of the system is provided (case I), passive systems where no external input is present at all (case II), and passive systems but with prior Lagrangian knowledge of a simpler system that forms a constituent of the system is provided (case III). The summary of the problem formulation is shown in Fig. 1.

In case I, by substituting Eq. (1) into Eq. (2) yields

3 $$\begin{aligned}{}&{\varvec{\tau }}_{pred} = \frac{d}{dt}\sum _{k=1}^{p}c_k \nabla _{\dot{{\varvec{q}}}}\phi _{k} - \sum _{k=1}^{p}c_k \nabla _{{\varvec{q}}}\phi _{k}, \end{aligned}$$

where ${\varvec{\tau }}_{pred}$ is the predicted value of the external input ${\varvec{\tau }}_{ext}$ given a set of coefficients ${\varvec{c}} = (c_1, c_2, ..., c_p)$. The time derivative $\frac{d}{dt}$ can be expanded further using the chain rule, resulting in the terms $\dot{{\varvec{q}}}$ and $\ddot{{\varvec{q}}}$ expressed as

4 $$\begin{aligned} \begin{aligned} {\varvec{\tau }}_{pred}&=\left( \sum _{k=1}^{p}c_k \nabla ^\top _{\dot{{\varvec{q}}}}\nabla _{\dot{{\varvec{q}}}}\phi _{k}\right) \ddot{{\varvec{q}}} + \left( \sum _{k=1}^{p}c_k \nabla ^\top _{{\varvec{q}}}\nabla _{\dot{{\varvec{q}}}}\phi _{k}\right) \dot{{\varvec{q}}} -\left( \sum _{k=1}^{p}c_k \nabla _{{\varvec{q}}}\phi _{k}\right) \\&= \sum _{k=1}^{p}c_k\left( \nabla ^\top _{\dot{{\varvec{q}}}}\nabla _{\dot{{\varvec{q}}}}\phi _{k}\ddot{{\varvec{q}}} + \nabla ^\top _{{\varvec{q}}}\nabla _{\dot{{\varvec{q}}}}\phi _{k}\dot{{\varvec{q}}} - \nabla _{{\varvec{q}}}\phi _{k} \right) \\&= \sum _{k=1}^{p}c_k\left( \textbf{M}_{k}\ddot{{\varvec{q}}} + \textbf{N}_{k}\dot{{\varvec{q}}} - \textbf{O}_{k} \right) , \end{aligned} \end{aligned}$$

where $\textbf{M}_{k}=\nabla ^\top _{\dot{{\varvec{q}}}}\nabla _{\dot{{\varvec{q}}}}\phi _{k}$, $\textbf{N}_{k}=\nabla ^\top _{{\varvec{q}}}\nabla _{\dot{{\varvec{q}}}}\phi _{k}$, and $\textbf{O}_{k}=\nabla _{{\varvec{q}}}\phi _{k}$. The Lagrangian of the system can be obtained by minimizing the cost function $J({\varvec{c}}) = \Vert {\varvec{\tau }}_{ext} - {\varvec{\tau }}_{pred}({\varvec{c}}) \Vert _{2}^{2}$.

However, in the case of where no external input is present (case II), we will just end up minimizing the residual cost function $J({\varvec{c}}) = \Vert - {\varvec{\tau }}_{pred}({\varvec{c}}) \Vert _{2}^{2}$. It can be observed from Eq. (4) that ${\varvec{\tau }}_{pred}({\varvec{c}})$ is in the form of a linear combination of coefficients ${\varvec{c}}$. Thus, the minimization of this residual cost function is equivalent to the determination of a sparse null space, which is an arduous task when using current optimization methods.

Instead, Eq. (4) can be modified so that we can solve for $\ddot{{\varvec{q}}}_{pred}$, which is expressed as

5 $$\begin{aligned} {\varvec{0}} &= \sum _{k=1}^{p}c_k\left( \textbf{M}_{k}\ddot{{\varvec{q}}} + \textbf{N}_{k}\dot{{\varvec{q}}} - \textbf{O}_{k} \right) ,\\ &\quad -\left( \sum _{k=1}^{p}c_k\textbf{M}_{k}\right) \ddot{{\varvec{q}}} = \sum _{k=1}^{p}c_k\left( \textbf{N}_{k}\dot{{\varvec{q}}} - \textbf{O}_{k} \right) ,\\ \quad \ddot{{\varvec{q}}}_{pred} & = \left( -\sum _{k=1}^{p}c_k\textbf{M}_{k}\right) ^{-1} \sum _{k=1}^{p}c_k\left( \textbf{N}_{k}\dot{{\varvec{q}}} - \textbf{O}_{k} \right) , \end{aligned}$$

where $\ddot{{\varvec{q}}}_{pred}$ represents the predicted value of the acceleration $\ddot{{\varvec{q}}}$ and $\left( \cdot \right) ^{-1}$ represents the matrix inverse. In practice, the Moore-Penrose pseudo-inverse^32^ is used to calculate Eq. (5) to avoid numerical instability. The cost function $J({\varvec{c}}) = \Vert \ddot{{\varvec{q}}} - \ddot{{\varvec{q}}}_{pred}({\varvec{c}}) \Vert _{2}^{2}$ is defined to learn the Lagrangian of the system.

**Figure 2:**
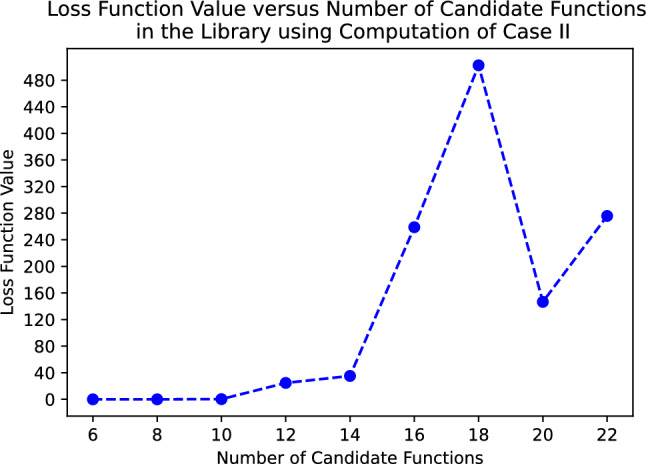
The value of loss function versus the number of candidate functions in the library for a double pendulum after 500 epochs of learning using the computation described in case II (non-convex loss function).

**Figure 3:**
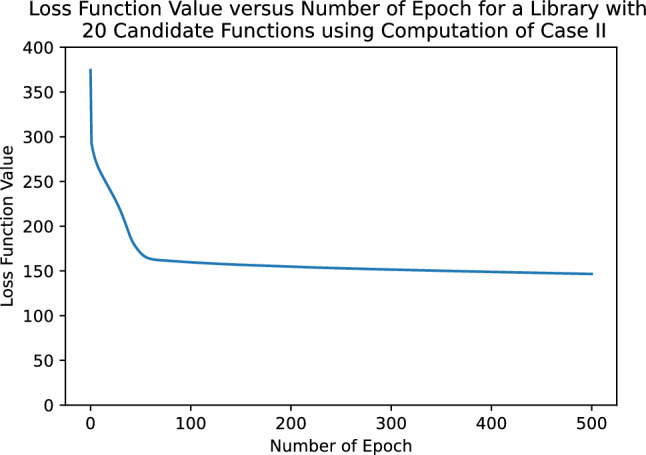
The value of loss function versus the number of epoch iteration for a double pendulum with 20 candidate functions using the computation described in case II (non-convex loss function).

Owing to the inverse operation, the cost function is non-convex with respect to the variable ${\varvec{c}}$, which means that the optimization process does not always converge to the global minimum. We empirically found that there is a tendency where more candidate functions correlate to a learning process that hardly converges. For example, we tested this computation method to discover a double pendulum with different numbesr of candidate functions in the library. As it can be seen in Fig. 2, even only at 12 candidate functions, the value of the loss function did not even reach a single digit even after 500 epochs. At 20 candidate functions as shown in Fig. 3, the loss function hardly decreased even following a lengthy iteration suggesting that the learning process converged to a local minimum . Therefore, this case is only used when no prior knowledge is available and the system is not complex, such as in a single pendulum. It is preferable for the external input ${\varvec{\tau }}_{ext}$ to be provided for more complex systems, such as a multidegree-of-freedom system. When no external input is provided, prior Lagrangian knowledge of a simpler system that forms a constituent of the larger system can be used to boost the learning process (case III) which will be explained in the following paragraph.

The Lagrangian for multidegree-of-freedom nonrelativistic systems can be described as $\mathcal {L} = \sum _i T_i - \sum _i V_i = \sum _i \left( T_i - V_i \right)$,where $T_i$ and $V_i$ are the kinetic and potential energies of each constituent of the system, respectively. As the total Lagrangian of the system is the sum of the Lagrangians of its constituents, it is reasonable to assume that the nonlinear terms that appear in each constituent also appear in the total Lagrangian of the system^30^.

Given the prior knowledge of a constituent of the system, one of the several terms that appear in the total Lagrangian of the system is selected and labeled as $\phi _{l}({\varvec{q}},\dot{{\varvec{q}}})$. The Lagrangian of a system is not unique; many forms of the Lagrangian can satisfy the Euler-Lagrange equation for a particular system. For example, $\mathcal {L}' = k\mathcal {L}$, where *k* is a constant, satisfies the Euler-Lagrange equation. By multiplying Eq. (1) with $k=\frac{1}{c_l}$, Eq. (1) can be modified as follows:

6 $$\begin{aligned}{}&\mathcal {L} = \phi _l({\varvec{q}},\dot{{\varvec{q}}}) + \sum \limits _{\begin{array}{c} k=1 \\ k\ne l \end{array}}^{p} c'_k\phi _{k}({\varvec{q}},\dot{{\varvec{q}}}), \end{aligned}$$

where $c'_k = \frac{c_k}{c_l}$. At this point, the variable $c'_k$ becomes the coefficient of interest. We can redefine $c_k:= c'_k$ for simplicity of notation. The Euler-Lagrange equation of the system can be expressed as

7 $$\begin{aligned} \begin{aligned}{}&-\frac{d}{dt} \nabla _{\dot{{\varvec{q}}}} \phi _l + \nabla _{{\varvec{q}}} \phi _l = \frac{d}{dt}\sum \limits _{\begin{array}{c} k=1 \\ k\ne l \end{array}}^{p}c_k \nabla _{\dot{{\varvec{q}}}}\phi _{k} - \sum \limits _{\begin{array}{c} k=1 \\ k\ne l \end{array}}^{p}c_k \nabla _{{\varvec{q}}}\phi _{k}. \end{aligned} \end{aligned}$$

We define the following notation:

8 $$\begin{aligned} {\varvec{\Upsilon }}_{right}&= \frac{d}{dt}\sum \limits _{\begin{array}{c} k=1 \\ k\ne l \end{array}}^{p}c_k \nabla _{\dot{{\varvec{q}}}}\phi _{k} - \sum \limits _{\begin{array}{c} k=1 \\ k\ne l \end{array}}^{p}c_k \nabla _{{\varvec{q}}}\phi _{k} \nonumber \\&=\sum \limits _{\begin{array}{c} k=1 \\ k\ne l \end{array}}^{p}c_k\left( \textbf{M}_{k}\ddot{{\varvec{q}}} + \textbf{N}_{k}\dot{{\varvec{q}}} - \textbf{O}_{k} \right) , \end{aligned}$$

9 $$\begin{aligned} {\varvec{\Upsilon }}_{left}&= -\frac{d}{dt} \nabla _{\dot{{\varvec{q}}}} \phi _l + \nabla _{{\varvec{q}}} \phi _l \nonumber \\&= -\textbf{M}_{l}\ddot{{\varvec{q}}} - \textbf{N}_{l}\dot{{\varvec{q}}} + \textbf{O}_{l}, \end{aligned}$$

where ${\varvec{\Upsilon }}_{left}$ and ${\varvec{\Upsilon }}_{right}$ represent the left-hand side and right-hand side of Eq. (7), respectively. It is possible to obtain the true Lagrangian of the system by minimizing the cost function $J({\varvec{c}}) =\Vert {\varvec{\Upsilon }}_{left} - {\varvec{\Upsilon }}_{right}({\varvec{c}}) \Vert _{2}^{2}$. In general, more than one option is available for $\phi _l$ to construct ${\varvec{\Upsilon }}_{left}$. In practice, all of these must be tested individually, and the one yielding the best model must be selected.

**Figure 4:**
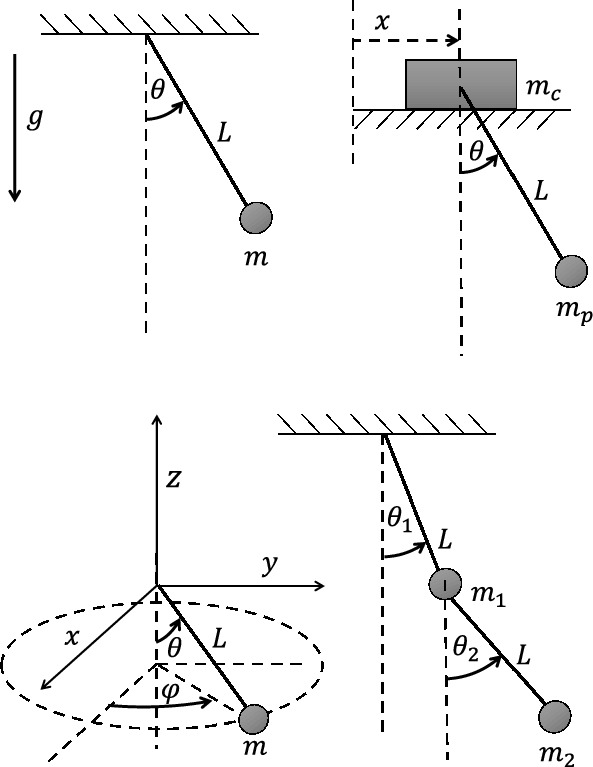
Dynamical systems used to verify xL-SINDy. From upper left to bottom right: A single pendulum, a cart pendulum, a spherical pendulum, and a double pendulum. For all systems, the length of the rod is $L = 1.0$ m, the mass of all pendulums except for the cart pendulum are $m = m_1 = m_2 = 1.0$ kg, and the gravitational acceleration is $g = 9.81$ m/s$^2$. For the cart-pendulum, the mass of the cart is $m_c = 1$ kg and the mass of the pendulum is $m_p = 0.5$ kg.

### Performance verification on different dynamical systems

We evaluated xL-SINDy using four ideal dynamical systems, as illustrated in Fig. 4. First, we tested xL-SINDy when the dynamical systems were excited by external inputs ${\varvec{\tau }}_{ext} = {\varvec{f}}(\sin {\omega t}, \cos {\omega t})$, where $\omega$ is a random frequency. The process that was used for the model learning is described by the computation of case I.

Furthermore, we tested the case of passive systems in which no external input ${\varvec{\tau }}_{ext}$ was provided. For the single pendulum, we assumed that no prior knowledge was available. Therefore, we used the computation that is described for case II. The systems of the cart pendulum, double pendulum, and spherical pendulum have a single pendulum as one of their constituents or are more complex versions of a single pendulum. Thus, assuming that the Lagrangian of the single pendulum had already been obtained, we could use the computation described for case III to bootstrap the learning process.

We collected the training data for each system by performing a simulation with 100 initial conditions for a period of 5 s each and a measurement frequency of 100 Hz. The initial conditions for all experiments were sampled under a uniform distribution between a certain threshold for each dynamical system. After obtaining the analytical form of the Lagrangian, we created a validation dataset to test the obtained model by calculating the predicted states for accuracy evaluation. We computed the Euler-Lagrange equation using the obtained model, retrieved the differential equation of the system, and integrated the equations for comparison with the actual validation data. Moreover, we tested the proposed method using training data that were corrupted by zero-mean white Gaussian noise $\mathcal {N}(0, \sigma )$ on magnitudes of different scales in the range of $10^{-8}<= \sigma <= 10^{-1}$. In the case of active systems, the Gaussian noise is only added to the measurement of state variables of the system. We assume that the function of the external inputs over time is known such as the case when external inputs are given from a computer. Finally, we compared the performance of xL-SINDy on several passive dynamical systems with noisy training data to that of SINDy-PI^33^.

**Figure 5:**
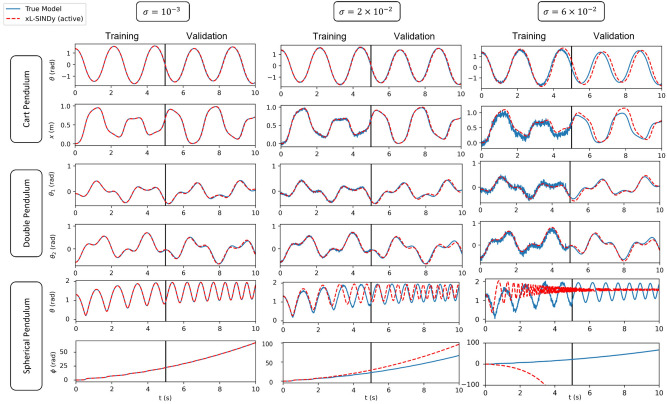
Comparison against true model when external excitation is provided. Training data consists of 100 initial conditions in a time period of 5 s each. Validation (extrapolation beyond the training data set) is conducted for 5 s afterward. The results shown are taken randomly from one of the initial conditions from the training data set for the cart pendulum, double pendulum, and spherical pendulum.

**Figure 6:**
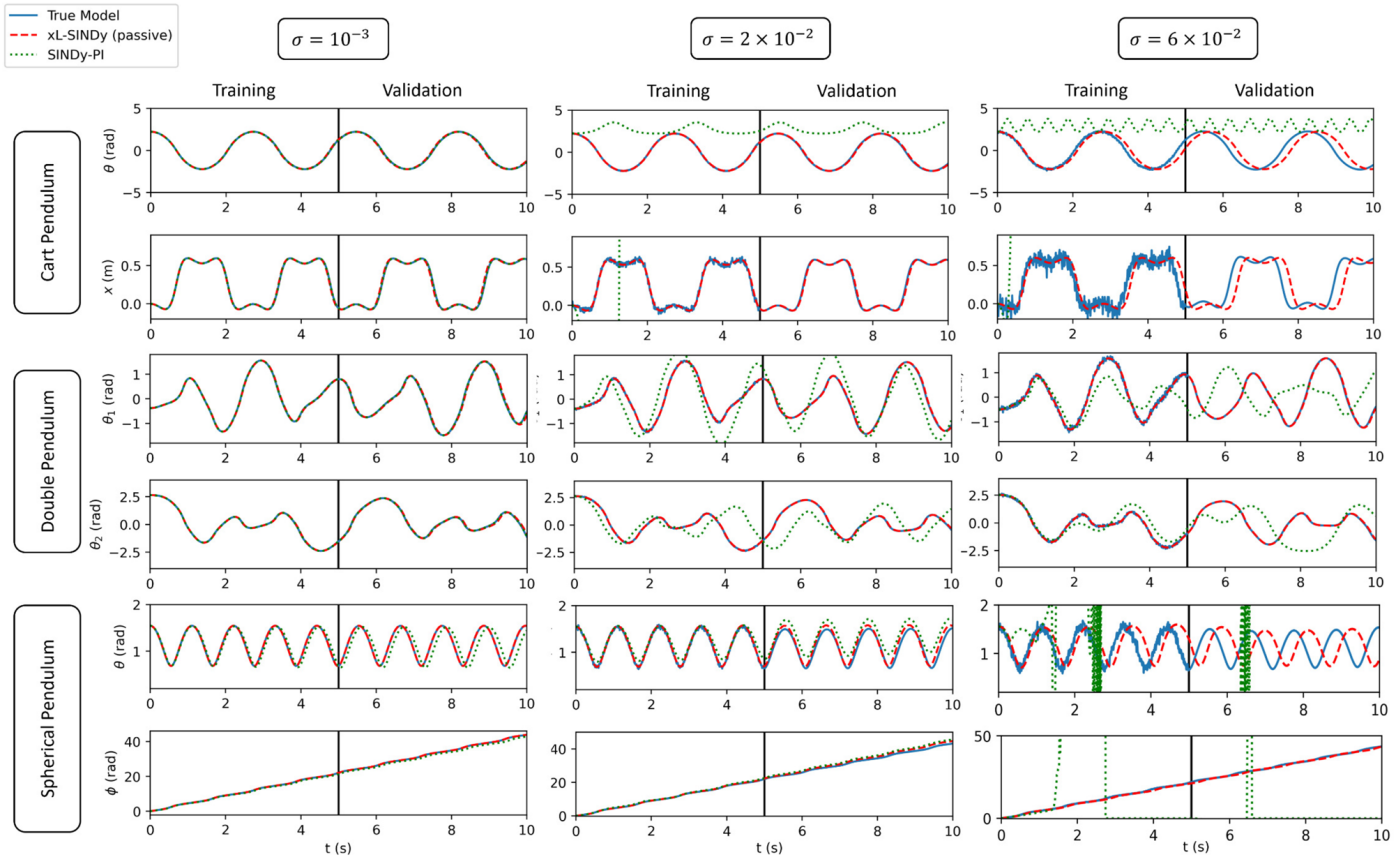
Comparison against true model and SINDy-PI when no external excitation is provided. Training data consists of 100 initial conditions in a time period of 5 s each. Validation (extrapolation beyond the training data set) is conducted for 5 s afterward. The results shown are taken randomly from one of the initial conditions from the training data set for the cart pendulum, double pendulum, and spherical pendulum.

The Lagrangian values that were obtained for each system are summarized in Table 1 for the active systems and in Table 2 for the passive systems. The performance of xL-SINDy compared to the true model in our simulation experiments on a cart pendulum, double pendulum, and spherical pendulum is presented at Fig. 5 for the active systems and at Fig. 6 for the passive systems, along with the comparison with SINDy-PI and for different noise levels. It can be observed from Tables 1 and 2 that xL-SINDy exhibited better performance in extracting the correct structure when external inputs were provided. It included fewer incorrect additional terms in the model than when no external input was provided.

Regarding the performance of xL-SINDy compared to SINDy-PI, presented in the second column of the plot in Fig. 6, when the noise magnitude was $\sigma = 2\times 10^{-2}$, it can be observed that SINDy-PI already started to deviate from the true models in all three dynamical systems. xL-SINDy still predicted accurate models at the same noise magnitude. It should also be noted that the model estimate of xL-SINDy was reasonable despite incorrect additional terms being included in the Lagrangian, as can be observed from the example of the cart pendulum under a noise magnitude of $\sigma = 6\times 10^{-2}$. This indicates that the model estimate is potentially usable, even when an incorrect Lagrangian structure is discovered.

**Table 1:** Extracted Lagrangian when external inputs are provided.

Noise magnitude	Single pendulum	Cart pendulum	Double pendulum	Spherical pendulum
True model	$$\begin{aligned}{}&0.500\dot{\theta }^2 + 9.810\cos {\theta } \end{aligned}$$	$$\begin{aligned}{}&0.250\dot{\theta }^2 + 0.750\dot{x}^2\\&{+}\,0.500\dot{x}\dot{\theta }\cos {\theta } \\&{+}\, 4.905\cos {\theta } \end{aligned}$$	$$\begin{aligned}{}&19.620\cos {\theta _1} + 9.810\cos {\theta _2} \\&{+}\,1.000\dot{\theta _1}\dot{\theta _2}\cos {\theta _1}\cos {\theta _2}\\&{+}\,1.000\dot{\theta _1}\dot{\theta _2}\sin {\theta _1}\sin {\theta _2}\\&{+}\,1.000\dot{\theta _1}^2+0.500\dot{\theta _2}^2 \end{aligned}$$	$$\begin{aligned}{}&0.500{\dot{\phi }^2\sin ^2{\theta }}+0.500{\dot{\theta }^2}\\ {}&{+}\,9.810\cos {\theta } \end{aligned}$$
$\sigma = 0$	$$\begin{aligned}{}&0.5\dot{\theta }^2 + 9.78\cos {\theta } \end{aligned}$$	$$\begin{aligned}{}&0.25\dot{\theta }^2 + 0.75\dot{x}^2\\&{+}\,0.5\dot{x}\dot{\theta }\cos {\theta } \\&{+}\,4.89\cos {\theta } \end{aligned}$$	$$\begin{aligned}{}&19.45\cos {\theta _1} + 9.72\cos {\theta _2} \\&{+}\,0.99\dot{\theta _1}\dot{\theta _2}\cos {\theta _1}\cos {\theta _2}\\&{+}\,0.99\dot{\theta _1}\dot{\theta _2}\sin {\theta _1}\sin {\theta _2}\\&{+}\,0.99\dot{\theta _1}^2+0.5\dot{\theta _2}^2 \end{aligned}$$	$$\begin{aligned}{}&0.5{\dot{\phi }^2\sin ^2{\theta }}+0.5{\dot{\theta }^2}\\ {}&{+}\,9.76\cos {\theta } \end{aligned}$$
$\sigma = 10^{-3}$	$$\begin{aligned}{}&0.5\dot{\theta }^2 + 9.78\cos {\theta } \end{aligned}$$	$$\begin{aligned}{}&0.25\dot{\theta }^2 + 0.75\dot{x}^2\\&{+}\,0.5\dot{x}\dot{\theta }\cos {\theta }\\&{+}\,4.88\cos {\theta } \end{aligned}$$	$$\begin{aligned}{}&19.31\cos {\theta _1} + 9.65\cos {\theta _2} \\&{+}\,0.99\dot{\theta _1}\dot{\theta _2}\cos {\theta _1}\cos {\theta _2}\\&{+}\,0.99\dot{\theta _1}\dot{\theta _2}\sin {\theta _1}\sin {\theta _2}\\&{+}\,0.99\dot{\theta _1}^2+0.49\dot{\theta _2}^2 \end{aligned}$$	$$\begin{aligned}{}&0.5{\dot{\phi }^2\sin ^2{\theta }}+0.5{\dot{\theta }^2}\\ {}&{+}\,9.8\cos {\theta } \end{aligned}$$
$\sigma = 2\times 10^{-2}$	$$\begin{aligned}{}&0.49\dot{\theta }^2 + 9.66\cos {\theta } \end{aligned}$$	$$\begin{aligned}{}&0.24\dot{\theta }^2 + 0.72\dot{x}^2\\&{+}\,0.48\dot{x}\dot{\theta }\cos {\theta }\\&{+}\,4.69\cos {\theta } \end{aligned}$$	$$\begin{aligned}{}&17.52\cos {\theta _1} + 8.76\cos {\theta _2} \\&{+}\,0.99\dot{\theta _1}\dot{\theta _2}\cos {\theta _1}\cos {\theta _2}\\&{+}\,0.89\dot{\theta _1}\dot{\theta _2}\sin {\theta _1}\sin {\theta _2}\\&{+}\,0.89\dot{\theta _1}^2+0.45\dot{\theta _2}^2 \end{aligned}$$	$$\begin{aligned}{}&0.31{\dot{\phi }^2\sin ^2{\theta }}+0.31{\dot{\theta }^2}\\ {}&{+}\,6.09\cos {\theta } \end{aligned}$$
$\sigma = 6\times 10^{-2}$	$$\begin{aligned}{}&0.44\dot{\theta }^2 + 8.73\cos {\theta } \end{aligned}$$	$$\begin{aligned}{}&0.17\dot{\theta }^2 + 0.53\dot{x}^2\\&{+}\,0.36\dot{x}\dot{\theta }\cos {\theta }\\&{+}\,3.32\cos {\theta } \end{aligned}$$	$$\begin{aligned}{}&10.72\cos {\theta _1} + 5.42\cos {\theta _2} \\&{+}\,0.55\dot{\theta _1}\dot{\theta _2}\cos {\theta _1}\cos {\theta _2}\\&{+}\,0.55\dot{\theta _1}\dot{\theta _2}\sin {\theta _1}\sin {\theta _2}\\&{+}\,0.55\dot{\theta _1}^2+0.27\dot{\theta _2}^2 \end{aligned}$$	$$\begin{aligned}{}&-0.66{\dot{\phi }^2\sin ^2{\theta }}-0.06{\dot{\theta }^2}\\ {}&{-}\,1.94\cos {\theta }\\&{\varvec{{+}\,0.08\dot{\theta }\dot{\phi }}}\\&{\varvec{{-}\,0.03\phi ^2}}^* \end{aligned}$$
$\sigma = 10^{-1}$	$$\begin{aligned}{}&0.36\dot{\theta }^2 + 7.19\cos {\theta } \\ \end{aligned}$$	$$\begin{aligned}{}&0.11\dot{\theta }^2 + 0.36\dot{x}^2 + \\&0.24\dot{x}\dot{\theta }\cos {\theta } \\&{+}\,2.03\cos {\theta } \\ \end{aligned}$$	$$\begin{aligned}{}&5.18\cos {\theta _1} + 2.66\cos {\theta _2} \\&{+}\,0.26\dot{\theta _1}\dot{\theta _2}\cos {\theta _1}\cos {\theta _2}\\&{+}\,0.26\dot{\theta _1}\dot{\theta _2}\sin {\theta _1}\sin {\theta _2}\\&{+}\,0.26\dot{\theta _1}^2+0.13\dot{\theta _2}^2 \end{aligned}$$	$$\begin{aligned}{}&0.04{\dot{\phi }^2\sin ^2{\theta }}+0.04{\dot{\theta }^2}\\ {}&{+}\,1.59\cos {\theta }\\&{\varvec{{-}\,0.1\dot{\theta }\phi \sin {\theta }}} \\&{\varvec{{-}\,0.22\phi ^2}}^* \end{aligned}$$

All results are obtained with the computation described in case I.

*Terms highlighted with bold font are extra terms that are not supposed to be included in the Lagrangian.

All numbers are rounded to 3 decimal places.

**Table 2:** Extracted Lagrangian when no external input is provided.

Noise magnitude	Single pendulum	Cart pendulum	Double pendulum	Spherical pendulum
True Model	$$\begin{aligned}{}&0.500\dot{\theta }^2 + 9.810\cos {\theta } \end{aligned}$$	$$\begin{aligned}{}&0.250\dot{\theta }^2 + 0.750\dot{x}^2\\&{+}\,0.500\dot{x}\dot{\theta }\cos {\theta } \\&{+}\, 4.905\cos {\theta } \end{aligned}$$	$$\begin{aligned}{}&19.620\cos {\theta _1} + 9.810\cos {\theta _2} \\&{+}\,1.000\dot{\theta _1}\dot{\theta _2}\cos {\theta _1}\cos {\theta _2}\\&{+}\,1.000\dot{\theta _1}\dot{\theta _2}\sin {\theta _1}\sin {\theta _2}\\&{+}\,1.000\dot{\theta _1}^2+0.500\dot{\theta _2}^2 \end{aligned}$$	$$\begin{aligned}{}&0.500{\dot{\phi }^2\sin ^2{\theta }}+0.500{\dot{\theta }^2}\\ {}&{+}\,9.810\cos {\theta } \end{aligned}$$
$\sigma = 0$	$$\begin{aligned}{}&0.295\dot{\theta }^2 + 5.797\cos {\theta } \end{aligned}$$	$$\begin{aligned}{}&1.000\dot{\theta }^2 + 2.975\dot{x}^2\\&{+}\,1.984\dot{x}\dot{\theta }\cos {\theta } \\&{+}\,19.755\cos {\theta } \end{aligned}$$	$$\begin{aligned}{}&19.620\cos {\theta _1} + 9.750\cos {\theta _2} \\&{+}\,1.000\dot{\theta _1}\dot{\theta _2}\cos {\theta _1}\cos {\theta _2}\\&{+}\,0.999\dot{\theta _1}\dot{\theta _2}\sin {\theta _1}\sin {\theta _2}\\&{+}\,1.000\dot{\theta _1}^2+0.499\dot{\theta _2}^2 \end{aligned}$$	$$\begin{aligned}{}&1.000{\dot{\phi }^2\sin ^2{\theta }}+1.000{\dot{\theta }^2}\\ {}&{+}\,19.630\cos {\theta } \end{aligned}$$
$\sigma = 10^{-3}$	$$\begin{aligned}{}&0.268\dot{\theta }^2 + 5.252\cos {\theta } \end{aligned}$$	$$\begin{aligned}{}&1.000\dot{\theta }^2 + 2.975\dot{x}^2\\&{+}\,1.984\dot{x}\dot{\theta }\cos {\theta }\\&{+}\,19.756\cos {\theta } \end{aligned}$$	$$\begin{aligned}{}&19.508\cos {\theta _1} + 9.755\cos {\theta _2} \\&{+}\,1.000\dot{\theta _1}\dot{\theta _2}\cos {\theta _1}\cos {\theta _2}\\&{+}\,0.999\dot{\theta _1}\dot{\theta _2}\sin {\theta _1}\sin {\theta _2}\\&{+}\,1.000\dot{\theta _1}^2+0.499\dot{\theta _2}^2 \end{aligned}$$	$$\begin{aligned}{}&1.000{\dot{\phi }^2\sin ^2{\theta }}+1.000{\dot{\theta }^2}\\ {}&{+}\,19.630\cos {\theta } \end{aligned}$$
$\sigma = 2\times 10^{-2}$	$$\begin{aligned}{}&0.334\dot{\theta }^2 + 6.540\cos {\theta } \end{aligned}$$	$$\begin{aligned}{}&1.000\dot{\theta }^2 + 2.993\dot{x}^2\\&{+}\,1.994\dot{x}\dot{\theta }\cos {\theta }\\&{+}\,19.534\cos {\theta } \end{aligned}$$	$$\begin{aligned}{}&19.545\cos {\theta _1} + 9.770\cos {\theta _2} \\&{+}\,1.000\dot{\theta _1}\dot{\theta _2}\cos {\theta _1}\cos {\theta _2}\\&{+}\,0.999\dot{\theta _1}\dot{\theta _2}\sin {\theta _1}\sin {\theta _2}\\&{+}\,1.000\dot{\theta _1}^2+0.499\dot{\theta _2}^2 \end{aligned}$$	$$\begin{aligned}{}&1.000{\dot{\phi }^2\sin ^2{\theta }}+1.000{\dot{\theta }^2}\\ {}&{+}\,19.600\cos {\theta } \end{aligned}$$
$\sigma = 6\times 10^{-2}$	$$\begin{aligned}{}&0.557\dot{\theta }^2 + 10.938\cos {\theta } \end{aligned}$$	$$\begin{aligned}{}&1.000\dot{\theta }^2 + 1.696\dot{x}^2\\&{+}\,1.136\dot{x}\dot{\theta }\cos {\theta }\\&{+}\,18.082\cos {\theta } \\ {}&{\varvec{{-}\,0.121\dot{x}^2\cos {\theta }}} \\&{\varvec{{+}\,1.463\cos ^3{\theta }}}^* \end{aligned}$$	$$\begin{aligned}{}&19.541\cos {\theta _1} + 9.753\cos {\theta _2} \\&{+}\,0.999\dot{\theta _1}\dot{\theta _2}\cos {\theta _1}\cos {\theta _2}\\&{+}\,0.999\dot{\theta _1}\dot{\theta _2}\sin {\theta _1}\sin {\theta _2}\\&{+}\,1.000\dot{\theta _1}^2+0.496\dot{\theta _2}^2 \end{aligned}$$	$$\begin{aligned}{}&0.130{\dot{\phi }^2\sin ^2{\theta }}+1.000{\dot{\theta }^2}\\ {}&{+}\,2.350\cos {\theta }\\ {}&{\varvec{{-}\,0.790\dot{\theta }^2\sin {\theta }}}\\&{\varvec{{-}\,0.430\dot{\theta }^2\cos {\theta }}}^* \end{aligned}$$
$\sigma = 10^{-1}$	$$\begin{aligned}{}&0.085\dot{\theta }^2 + 1.540\cos {\theta } \\&{\varvec{{-}\,0.129\dot{\theta }\sin {\theta }}} \\&{\varvec{{+}\,0.551\sin ^2{\theta }}}\\&{\varvec{{-}\,0.019\theta ^2}}^* \end{aligned}$$	$$\begin{aligned}{}&1.000\dot{\theta }^2 + 1.562\dot{x}^2 + \\&1.050\dot{x}\dot{\theta }\cos {\theta } \\&{+}\,19.504\cos {\theta } \\&{\varvec{{-}\,0.143\dot{\theta }^2\cos {\theta }}}^* \end{aligned}$$	$$\begin{aligned}{}&19.381\cos {\theta _1} + 9.679\cos {\theta _2} \\&{+}\,0.998\dot{\theta _1}\dot{\theta _2}\cos {\theta _1}\cos {\theta _2}\\&{+}\,0.992\dot{\theta _1}\dot{\theta _2}\sin {\theta _1}\sin {\theta _2}\\&{+}\,1.000\dot{\theta _1}^2+0.495\dot{\theta _2}^2 \end{aligned}$$	$$\begin{aligned}{}&-0.2{\dot{\phi }^2\sin ^2{\theta }}+1.000{\dot{\theta }^2}\\ {}&{+}\,5.12\cos {\theta }\\&{\varvec{{-}\,1.100\dot{\theta }^2\sin {\theta }}} \\&{\varvec{{-}\,0.560\dot{\theta }^2\cos {\theta }}} \\&{\varvec{{-}\,0.055\dot{\phi }^2\sin {2\theta }}}^* \end{aligned}$$

For the single pendulum, the result is obtained with a computation described by case II, while the rest are obtained with a computation described by case III with the knowledge of the Lagrangian of a single pendulum.

*Terms highlighted with bold font are extra terms that are not supposed to be included in the Lagrangian.

All numbers are rounded to 3 decimal places.

#### Single pendulum

In an active system, the correct Lagrangian structure–one without additional or missing terms compared to the true Lagrangian form–could be obtained in the presence of a noise magnitude of up to $\sigma = 1\times 10^{-1}$. In a passive system, the correct Lagrangian structure could be obtained with a noise magnitude of up to $\sigma = 6\times 10^{-2}$. Although the obtained coefficients differed from those of the true model, the ratio of the coefficients between the two terms was close compared to the true model.

#### Cart pendulum

According to Tables 1 and 2, xL-SINDy could recover the correct structure of the Lagrangian in the presence of a noise magnitude of up to $\sigma = 1\times 10^{-1}$ with external input and a noise magnitude up to $\sigma = 4\times 10^{-2}$ without external input. In contrast, SINDy-PI could only recover the correct structure with a noise magnitude of up to $\sigma = 5\times 10^{-3}$. Therefore, xL-SINDy was eight times more robust than SINDy-PI in the presence of noise for the cart pendulum. Moreover, SINDy-PI sometimes predicted a model that blew up with a large noise magnitude, as indicated in the second column of Fig. 6. This is because it predicted the denominator terms incorrectly. Unlike SINDy-PI, xL-SINDy addresses the Lagrangian instead of the actual equation of motion. As there is no denominator in the Lagrangian, xl-SINDy still provides reasonable predictions, even though incorrect additional terms are included in the Lagrangian.

#### Double pendulum

According to the experimental results, xL-SINDy identified the correct structure with a noise magnitude of up to $\sigma = 10^{-1}$ in both active and passive cases, whereas SINDy-PI could extract the correct structure of the equations of motion with a noise magnitude of only up to $\sigma = 10^{-2}$. Hence, xL-SINDy was 10 times more robust against noise than SINDy-PI in this experiment. As can be observed from the summary table, xL-SINDy was quite robust for the double pendulum compared to the other dynamical systems. A possible reason that xL-SINDy was more robust for the double pendulum is the chaotic signal that is caused by the double pendulum. The double pendulum yields an entirely different signal path for every initial condition owing to its inherently chaotic nature, thereby creating rich training data.

#### Spherical pendulum

In this experiment, xL-SINDy was robust up to $\sigma = 2 \times 10^{-2}$ for both the active and passive system cases. In contrast, SINDy-PI was only robust up to $\sigma = 1 \times 10^{-3}$. Thus, xL-SINDy was 20 times more robust against noise than SINDy-PI. It can be observed from the second column of Fig. 5 and Table 2 that, although the correct structure could be obtained for the spherical pendulum at $\sigma = 2 \times 10^{-2}$, the performance of xL-SINDy was inferior for an active system. This is because in a passive system, a Lagrangian that is multiplied by a constant is still a valid Lagrangian. Thus, provided that the ratio between each coefficient is the same as that of the true model, the obtained model is also correct. However, in an active system, the Lagrangian is unique because an external constraint exists. Therefore, although the correct structure is obtained, if the coefficients do not closely match those of the true model, a less accurate long-term prediction ability will be exhibited.

## Discussion

It can be concluded from the simulation results that xL-SINDy is more robust in obtaining the correct Lagrangian structure if an external input is provided to the system. Although xL-SINDy can obtain the correct Lagrangian structure in the presence of greater noise, it does not guarantee accurate long-term prediction ability. This is because the Lagrangian of the system is unique when external input is provided; thus, mismatched coefficients will result in a deviation in the long-term prediction. In contrast, this will not be a problem if no external input is provided because the Lagrangian is not unique. Provided that the ratio between each coefficient is close to that of the true model, an effective long-term prediction model can still be established. However, in the absence of an external input, xL-SINDy is slightly less robust in determining the correct Lagrangian structure.

**Figure 7:**
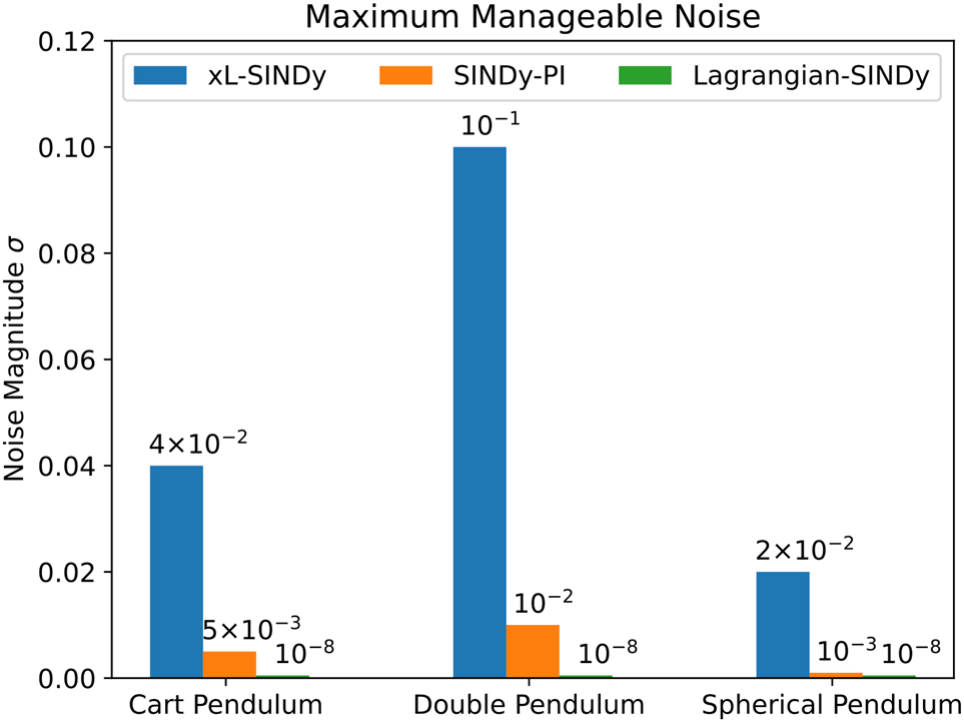
Comparison of the maximum level of manageable noise between xL-SINDy, Lagrangian-SINDy, and SINDy-PI. The maximum manageable noise is defined as the maximum noise level before an incorrect model structure is discovered. xL-SINDy provides the most robust performance against noisy training data in all simulations of three different dynamical systems.

We used passive cases as the baseline for xL-SINDy to compare it with other models. A comparison of xL-SINDy, SINDy-PI, and Lagrangian-SINDy is presented in Fig. 7. The experimental results demonstrate that xL-SINDy outperformed the other methods in terms of noise robustness in all three dynamical systems that were used for comparison. Moreover, xL-SINDy can overcome the challenges faced by Lagrangian-SINDy; xL-SINDy can discover the correct Lagrangian expression for idealized nonlinear dynamical systems in the presence of a substantially higher noise magnitude. Furthermore, xL-SINDy successfully extracts the Lagrangian in cases where Lagrangian-SINDy fails to do so, such as the non-actuated spherical pendulum^30^. Although the obtained coefficients may not be exactly the same as those of the true models, the ratio between the coefficients of each term is close to that of the true models.

There is a couple of reasons why xL-SINDy is much more robust than Lagrangian-SINDy. As previously mentioned, Lagrangian-SINDy finds the Lagrangian through the total energy which coincides with the Hamiltonian in mechanical systems. Lagrangian-SINDy involves two different regression where it first compares the change of total energy with the external input of the systems, and extract Lagrangian through reverse Legendre transformation. In contrast, xL-SINDy directly finds the Lagrangian through the Euler-Lagrange equation which is straightforward. Furthermore, to find the sparse solution, Lagrangian-SINDy used a combinatorial search of all possible subsets of regression in the case of active systems and Singular Value Decomposition (SVD) in the case of passive systems. Since no regularization is enforced in the optimization methods, the results are more sensitive to noise. On the other hand, xL-SINDy is formulated so that the optimization problem is a convex problem, thus finding a sparse solution can be done using the convex optimization method with $\ell _1$ regularization.

Although SINDy-PI is also found to be robust against noise of up to a certain magnitude, xL-SINDy is 8 to 20 times more robust against noise. SINDy-PI attempts to seek an expression of dynamics that may contain rational functions. For this purpose, SINDy-PI reformulates the problem into an implicit form and it requires the library to include candidate functions of the states and the time derivative of the system states. Consequently, SINDy-PI must also include the ${\varvec{q}}$, $\dot{{\varvec{q}}}$, and $\ddot{{\varvec{q}}}$ variables to construct terms in the library. Unlike SINDy-PI, which deals with the actual equation of motion, xL-SINDy deals with the Lagrangian that only requires the ${\varvec{q}}$ and $\dot{{\varvec{q}}}$ variables to construct the terms in the library. Hence, xL-SINDy naturally contains fewer terms in the library to obtain the same order of a family function (e.g., second order of polynomial functions).

A further advantage of xL-SINDy over SINDy-PI is that xL-SINDy can provide greater coverage of various terms with fewer coefficient parameters in the actual equation of motion. This is owing to the inherent nature of the Lagrangian formulation. For example, let us consider a Lagrangian that contains only one term, which is expressed by

10 $$\begin{aligned} \mathcal {L} = c_0\phi _0({\varvec{q}}, \dot{{\varvec{q}}}). \end{aligned}$$

If we substitute this into the Euler-Lagrange formula, we obtain an equation of motion that contains three different terms, as follows:

11 $$\begin{aligned} \begin{aligned} {\varvec{0}}&=\left( c_0 \nabla ^\top _{\dot{{\varvec{q}}}}\nabla _{\dot{{\varvec{q}}}}\phi _{0}\right) \ddot{{\varvec{q}}} + \left( c_0 \nabla ^\top _{{\varvec{q}}}\nabla _{\dot{{\varvec{q}}}}\phi _{k}\right) \dot{{\varvec{q}}} \\&\quad -\left( c_0 \nabla _{{\varvec{q}}}\phi _{k}\right) \\&= c_0\left( \nabla ^\top _{\dot{{\varvec{q}}}}\nabla _{\dot{{\varvec{q}}}}\phi _{k}\ddot{{\varvec{q}}} + \nabla ^\top _{{\varvec{q}}}\nabla _{\dot{{\varvec{q}}}}\phi _{k}\dot{{\varvec{q}}} - \nabla _{{\varvec{q}}}\phi _{k} \right) . \end{aligned} \end{aligned}$$

As can be observed from the above equation, although three different terms appear in the equation of motion, all of them correspond to the same coefficient $c_0$. This is not the case with SINDy-PI because it requires three different coefficients to represent the different terms in the equations of motion. Hence, it would be easier to learn the model using xL-SINDy than using SINDy-PI with fewer parameters, while maintaining the same amount of coverage of possible terms in the actual equations of motion. This is one reason that xL-SINDy is more robust than SINDy-PI.

Another reason that xL-SINDy is more robust than SINDy-PI is the performance of the learning process. SINDy-PI uses the sequential threshold least-squares method^33^, the basic concept of which is to run the least-squares method and remove terms with low coefficients sequentially (hard thresholding). However, we experimentally determined that the sequential least-squares method often fails to remove non-relevant terms if the training data are corrupted. Thus, the concept of hard thresholding from the sequential least-squares method was combined with soft thresholding from the Lasso regression with the proximal gradient method. It was found that this approach achieved better performance in removing non-relevant terms with a higher level of noise. As shown in Table 3, although xL-SINDy had more terms in the library than SINDy-PI, xL-SINDy still outperformed SINDy-PI with a higher level of noise for the double and spherical pendulums.

Finally, SINDy-PI may encounter a problem when an incorrect combination of the denominator terms is discovered. In a rational function, when the denominator is zero, its value blows up, which was the case for SINDy-PI for the cart and spherical pendulums according to our experimental results. In contrast, xL-SINDy only contains rational terms because it is a Lagrangian mechanical function. Therefore, it minimizes the possibility of the model blowing up owing to incorrect terms.

As with other learning-based methods, xL-SINDy introduces several hyperparameters during the learning process, such as the sparsity constraint $\lambda$, learning rate $\alpha$, tolerance for the cost function, and cut-off threshold in the hard-thresholding process. Hyperparameter tuning is also vital for the learning outcome, particularly the initial values of the learning rate $\alpha$ and sparsity constraint $\lambda$. At present, the hyperparameter tuning process is performed manually through trial and error.

One major limitation of xL-SINDy is the difficulty in designing the library. Prior knowledge of the systems is essential to deciding what candidate functions we should include in the library. A large number of candidate functions in the library are more likely to be sufficient, but it makes the sparse optimization more challenging and less robust against noise^33^. Hence, balancing this trade-off is crucial for the outcome of the learning process.

A better mechanism for handling a large library is necessary for applying xL-SINDy to more complex systems with a higher degree of freedom. One possible means of addressing a large number of libraries is the library-bootstrapping method, as in the case of Ensemble-SINDy^34^. In this method, many smaller libraries are created by sampling terms without replacement from the original library and several different models are learned separately. Once the learning process is complete, all terms with a low probability of inclusion are removed. This process can be repeated until sufficient results are obtained.

The presence of external non-conservative forces acting on the systems were not considered in this study. In real-world scenarios, non-conservative forces of varying degrees, such as damping or friction, are always present. The consideration of such external forces in the model is important to apply xL-SINDy to real systems. One major difficulty of incorporating non-conservative forces is that our model is based on the Lagrangian formula, and the Lagrangian formula does not take into account non-conservative forces. Thus, to include non-conservative forces, a different model of non-conservative forces need to be included in the final Euler-Lagrange equation. A possible means of incorporating non-conservative forces is to use the generalized Rayleigh dissipation function^35^. Similar to the Lagrangian, the Rayleigh dissipation function is a single scalar quantity that can be incorporated into the Euler-Lagrange equation. The generalized Rayleigh dissipation function can be modeled as a linear combination of candidate functions, and the Lagrangian and Rayleigh dissipation functions can be learned simultaneously. However, this will also mean that the number of candidate terms in the library and coefficient will be exceedingly large, and the extraction of sparse solutions with the presented optimization method in this paper is still challenging. Thus, we will consider handling non-conservative forces as our future work. In addition, our group works also on distributed neural integrator for inducing synchronized oscillations in unknown mechanical systems as non-while box approach^36^. The combination of while box modeling and black box modeling can be an interesting direction of the research.

**Figure 8:**
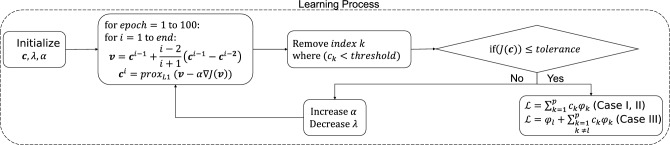
Diagram of the sparse regression using proximal gradient method. The Combination of soft threshold from proximal gradient method, and hard threshold at the end of the learning stage accelerates the learning process.

**Table 3:** Comparison of the size of the library.

Method	Dynamical system		
	Cart pendulum	Double pendulum	Spehrical pendulum
xL-SINDy	55	87	59
SINDy-PI	90	40	49

## Materials and methods

### Sparse regression with proximal gradient method

The proposed learning method for obtaining the Lagrangian is depicted in Fig. 8. The corresponding code is available from https://github.com/AdamPurnomo/Extended-Lagrangian-SINDy-xL-SINDy-. First, the problem was formulated, as described in the section called New Problem Formulation for Lagrangian Acquisition. Given a dynamic system, time-series data were gathered from several initial conditions regarding $\{t_i,{\varvec{q}}(t_{i}), \dot{{\varvec{q}}}(t_{i}), \ddot{{\varvec{q}})}(t_{i}, {\varvec{\tau }}_{ext}(t_{i})\}_{i=1}^{N}$. Subsequently, a library of candidate functions was constructed.

In general, the optimization problem becomes more difficult when the library of candidate functions is larger. This is particularly true when several candidate functions may behave in a similar manner, such as functions of the trigonometric family. It is important to construct a library that is sufficient but not excessively large so that the optimization problem remains tractable. However, it is also preferable not to include trivial terms that satisfy the Euler-Lagrange equation, regardless of the trajectories, such as $\mathcal {L} = {\varvec{q}}^n\dot{{\varvec{q}}}$. Technically, although trivial terms do not affect the system behavior, it is better to remove them so that unnecessary complexity in the library can be reduced. A different cost function should be defined depending on the case.

As mentioned previously, we believe that the correct solution is sparse, in which most coefficients are zero. Therefore, we added the $\ell _1$ regularization term to the cost function for the sparsity constraint^18^, which is expressed as

12 $$\begin{aligned} J'({\varvec{c}}) = J({\varvec{c}}) + \lambda \Vert {\varvec{c}}\Vert _1, \end{aligned}$$

where $\lambda$ is a sparsity-promoting parameter that must be carefully tuned. Several methods that can be used to solve LASSO include coordinate descent method^37^, sub-gradient methods^38^, proximal gradient method^39^, and Least-Angle Regression^40^. The accelerated proximal gradient descent method^39^ was used in this study to minimize the composite cost function that is defined above. Given an initial point ${\varvec{c}}^0$, the update step of the proximal gradient descent is defined as

13 $$\begin{aligned}{}&{\varvec{v}} = {\varvec{c}}^{i-1} + \frac{i-2}{i+1}\left( {\varvec{c}}^{i-1} - {\varvec{c}}^{i-2}\right) , \end{aligned}$$

14 $$\begin{aligned}{}&{\varvec{c}}^{i} = prox_{\ell _1}\left( {\varvec{v}} - \alpha \nabla J({\varvec{v}}) \right) , \end{aligned}$$

where ${c}^{i}$ is the coefficient ${\varvec{c}}$ at iteration *i*, $\alpha$ is the learning rate, and $prox_{\ell _1}(\cdot )$ is the proximal operator for the $\ell _1$ norm. The $\ell _1$ norm penalty term is a separable sum of the components of its input and a proximal operator is used to minimize this term. The proximal operator for the $\ell _1$ norm is well defined separately for each component of the input and is expressed as follows:

15 $$\begin{aligned}{}&[prox_{\ell _1}({\varvec{\beta }})]_{k} = \text {sign}(\beta _k)\max (|\beta _k|-\lambda ,0), \end{aligned}$$

where *k* is the $k{th}$ entry of input vector ${\varvec{\beta }}$. For case III, if other terms are known that appear in the Lagrangian but are not used to construct ${\varvec{\Upsilon }}_{left}$, we do not impose a penalty on these terms by not applying the proximal operator in Eq. (15) for the index *k* corresponding to these terms.

We initialized the values of the coefficient ${\varvec{c}}$, learning rate $\alpha$, and $\ell _1$ norm penalty parameter $\lambda$. The learning process was performed in several stages, with 100 epochs and a batch size of 128 for each stage, until the cost function reached the defined tolerance value, as illustrated in Fig. 1. The ideal tolerance value is $10^{-3}$. However, convergence to this value may not be possible in the presence of noise and the tolerance value must be relaxed; otherwise, the algorithm will never stop. In general, the number of candidate functions in the library is initially large, and we should attempt to eliminate as many non-relevant candidate functions as possible during the first learning stage. Therefore, we initially set the value of $\lambda$ to be quite high, between 1-5.

It is important to note that each candidate function may have a different magnitude scale. The $\ell _1$ norm penalizes all terms equally, regardless of the magnitude scale, resulting in more candidate functions with smaller magnitude scales being penalized. It may or may not be necessary to perform scaling in the first learning stage, where the value of $\lambda$ is high, by multiplying each candidate function by the scaling term $s_k$ in Eq. (1) for cases I and II, or Eq. (6) for case III, depending on the differences in the magnitude scale of each candidate function. Moreover, the learning rate is an important hyperparameter, particularly during the first learning stage. A high learning rate in the initial stage may cause the relevant terms to be penalized, thereby preventing the model from obtaining the true Lagrangian of the system. The learning rate was set to $\alpha <= 10^{-5}$ during the initial stage.

We performed hard thresholding at the end of every learning stage by removing the index *k* from Eq. (1) or (6), where $c_k < threshold$. This step effectively reduced the number of candidate functions that were considered in the learning process, which means that the convergence is much faster than when hard thresholding is not performed. Subsequently, we verified whether or not the cost function reached the tolerance. If this is the case, we proceed to the next learning stage. Owing to fewer candidate functions following the previous hard thresholding process, the value of $\lambda$ can be decreased and the learning rate $\alpha$ can be increased to accelerate the learning process. The rates of the increment $\alpha$ and decrement $\lambda$ are also significant. If $\alpha$ is increased too rapidly, the optimization process may take longer to converge, and if $\lambda$ is decreased too rapidly, it may not be possible to eliminate all non-relevant terms. A tuning process is required to determine the most appropriate rate for increasing $\alpha$ and decreasing $\lambda$. This step was repeated until the cost function reached the tolerance value. The learning process generally requires approximately three or four stages for the tolerance value to be reached. Once the tolerance value was reached, the value of the coefficient was computed using Eq. (1) for cases I and II or Eq. (6) for case III, and the analytical form of the Lagrangian of the system was obtained.

### Learning of single pendulum

The state of a single pendulum is described by $[\theta , \dot{\theta }]$, and the Lagrangian expression of a single pendulum is expressed by $\mathcal {L} = \frac{1}{2}m\dot{\theta }^2 + mg\cos {\theta }$. The true Lagrangian expression that was obtained by substituting the parameters in Fig. 4 is shown in the second row and second column of Table 1. We created a polynomial combination of $\{\theta , \dot{\theta }, \cos {\theta }, \sin {\theta }\}$ up to the second order, excluding trivial terms such as $\dot{\theta }$ and $\theta \dot{\theta }$, to construct a library of 12 candidate functions. Training data with the initial conditions of $[-\pi< \theta < \pi ,0]$ were created.

The initial values of the hyperparameters were $\alpha = 10^{-5}$ and $\lambda = 0.1$. The cut-off threshold was $10^{-2}$ for the initial learning stage and $10^{-1}$ for the subsequent learning stages. In the subsequent learning stages, $\alpha$ was increased by a factor of two and $\lambda$ was decreased by a factor of 10. The training converged in three stages for a noise magnitude of $\sigma <= 10^{-3}$ and four stages for higher magnitudes with a relaxed tolerance value.

### Learning of cart pendulum

The state of the cart pendulum is represented as $[\theta , \dot{\theta }, x, \dot{x}]$, and the Lagrangian with numerical coefficients that was obtained using the parameters in Fig. 4 is presented in the second row and third column of Table 1. A polynomial combination of $\{\dot{\theta }, \cos {\theta }, \sin {\theta }, x, \dot{x}\}$ up to the third order was constructed to create a library of 55 candidate functions. In this case, we excluded the term $\theta$ because it does not appear in the Lagrangian of a single pendulum system. Training data with the initial conditions of $[-\pi< \theta < \pi , 0, 0,0]$ were created.

The Lagrangian of a single pendulum contains $\dot{\theta }^2$ and $\cos {\theta }$. Hence, both terms also appear in the Lagrangian of the cart pendulum. Both $\dot{\theta }^2$ and $\cos {\theta }$ were tested to construct ${\varvec{\Upsilon }}_{left}$, as described in Eq. (9), and the term $\dot{\theta }^2$ yielded better results. The initial values of the hyperparameters were $\alpha = 10^{-5}$ and $\lambda = 1$. The cut-off threshold, increment of $\alpha$ and decrease in $\lambda$ were the same as in the previous case. The training converged in three stages for a noise magnitude of $\sigma <= 2\times 10^{-2}$ and four stages for higher magnitudes with a relaxed tolerance value.

### Learning of double pendulum

Given the state of a double pendulum, $[\theta _1, \theta _2, \dot{\theta _1}, \dot{\theta _2}]$ and the system parameters in Fig. 4, the expression of the Lagrangian with numerical coefficients is shown in the second row and fourth column of Table 1. We first separated the sets of trigonometric terms $\{\cos {\theta _1},\sin {\theta _1},\sin {\theta _1},\sin {\theta _2}\}$ and non-trigonometric terms $\{\dot{\theta _1},\dot{\theta _2}\}$ to construct a library of candidate functions. We created a polynomial combination of up to the second order for each set, resulting in 14 and 5 candidate functions, respectively. Subsequently, we generated cross-terms between the two sets, thereby creating 70 candidate functions, forming a total of 89 candidate functions in the library. Training data were created under the initial conditions of $[-\pi< \theta _1< \pi ,-\pi< \theta _2 < \pi ,0,0]$. Both constituents of the double pendulum are single pendulums. Hence, there were four options for construction. Both $\dot{\theta _1}^2$ and $\dot{\theta _2}^2$ yielded equally good results.

The results are presented in Tables 1 and 2 display the values with $\dot{\theta _1}^2$ that are used to construct ${\varvec{\Upsilon }}_{left}$. The initial values of the hyperparameters were $\alpha = 5\times 10^{-6}$ and $\lambda = 1$. The cut-off threshold, increment of $\alpha$ and decrease in $\lambda$ were the same as in the previous cases.

### Learning of spherical pendulum

The state of the spherical pendulum is represented as $[\theta , \phi , \dot{\theta }, \dot{\phi }]$, and the true Lagrangian expression is displayed in the second row and fifth column of Table 1. As in the case of the double pendulum, we first separated the trigonometric terms $\{\cos {\theta },\sin {\theta }\}$ and non-trigonometric terms $\{\dot{\theta },\phi ,\dot{\phi }\}$, created polynomial combinations for both sets up to the second order, and added cross-terms between the two sets. In total, the library contained 59 candidate functions. The training data were created with the initial conditions of $[\pi /3< \theta < \pi /2,0,0,\pi ]$. We deliberately selected high values of $\theta$ and $\dot{\phi }$ for the initial conditions because the equation of motion contained $\frac{1}{\sin {\theta }}$, which may blow up for a small value of $\theta$.

A spherical pendulum is a higher-dimensional analog of a single pendulum; therefore, we can consider the Lagrangian of a spherical pendulum as the sum of the Lagrangian of the pendulum in the $\hat{\theta }$ and $\hat{\phi }$ directions. We used $\dot{\theta }^2$ and $\cos {\theta }$ to construct ${\varvec{\Upsilon }}_{left}$ because the Lagrangian of a single pendulum in the $\hat{\theta }$ direction was already known. The initial values of the hyperparameters were $\alpha = 1\times 10^{-5}$ and $\lambda = 1$. The cut-off threshold, increment of $\alpha$ and decrease in $\lambda$ were the same as in all previous cases (Supplementary Information).

## Supplementary Information

Supplementary Video 1.

Supplementary Information 2.

Supplementary Information 3.

## Data Availability

All codes used to perform the analyses are available from the repository. https://github.com/AdamPurnomo/Extended-Lagrangian-SINDy-xL-SINDy-.

## References

[1] Popper KR (1934). The Logic of Scientific Discovery.

[2] Lecun Y, Bottou L, Bengio Y, Haffner P (1998). Gradient-based learning applied to document recognition. Proc. IEEE.

[3] Krizhevsky A, Sutskever I, Hinton GE (2012). ImageNet classification with deep convolutional neural networks. Adv. Neural Inf. Process. Syst..

[4] Battaglia, P., Pascanu, R., Lai, M., Jimenez Rezende, D. & kavukcuoglu, k. Interaction Networks for Learning about objects, relations and physics. *Adv. Neural Inf. Process. Syst.* **29**, (2016),

[5] Lenz, I., Knepper, R. & Saxena, A. DeepMPC: Learning deep latent features for model predictive control. In *Robotics: Science and Systems XI* (2015).

[6] Sahoo, S., Lampert, C. & Martius, G. Learning equations for extrapolation and control. In *Proceedings of the 35th International Conference on Machine Learning*, (eds Dy, J. & Krause, A.) vol. 80 of *Proceedings of Machine Learning Research*, 4442–4450 (PMLR, 2018).

[7] Greydanus, S., Dzamba, M. & Yosinski, J. Hamiltonian neural networks. *Adv. Neural Inf. Process. Syst.* **32**, (2019).

[8] Cranmer, M. *et al.*, Lagrangian neural networks (2020).

[9] Lusch B, Kutz JN, Brunton SL (2018). Deep learning for universal linear embeddings of nonlinear dynamics. Nat. Commun..

[10] Wehmeyer C, Noé F (2018). Time-lagged autoencoders: Deep learning of slow collective variables for molecular kinetics. J. Chem. Phys..

[11] Mardt A, Pasquali L, Wu H, Noé F (2018). VAMPnets for deep learning of molecular kinetics. Nat. Commun..

[12] Koopman BO, Neumann Jv (1932). Dynamical systems of continuous spectra. Proc. Natl. Acad. Sci..

[13] Mezić I, Banaszuk A (2004). Comparison of systems with complex behavior. Phys. D Nonlinear Phenomena.

[14] Cranmer, M. *et al.*, Discovering symbolic models from deep learning with inductive biases. In *Advances in Neural Information Processing Systems*, vol. 33 (eds Larochelle, H. *et al.*), 17429–17442 (Curran Associates, Inc., 2020).

[15] Xie T, France-Lanord A, Wang Y, Shao-Horn Y, Grossman JC (2019). Graph Dynamical Networks for unsupervised learning of atomic scale dynamics in materials. Nat. Commun..

[16] Schmidt M, Lipson H (2009). Distilling free-form natural laws from experimental data. Science.

[17] Brunton SL, Proctor JL, Kutz JN (2016). Discovering governing equations from data by sparse identification of nonlinear dynamical systems. Proc. Natl. Acad. Sci..

[18] Tibshirani R (1996). Regression shrinkage and selection via the lasso. J. R. Stat. Soc. Ser. B Methodol..

[19] Hastie T, Tibshirani R, Friedman J (2001). The Elements of Statistical Learning, Springer Series in Statistics.

[20] Sorokina M, Sygletos S, Turitsyn S (2016). Sparse identification for nonlinear optical communication systems: SINO method. Opt. Express.

[21] Dam M, Brøns M, Rasmussen JJ, Naulin V, Hesthaven JS (2017). Sparse identification of a predator-prey system from simulation data of a convection model. Phys. Plasmas.

[22] Loiseau JC, Brunton SL (2018). Constrained sparse Galerkin regression. J. Fluid Mech..

[23] de Silva BM, Higdon DM, Brunton SL, Kutz JN (2020). Discovery of physics from data: Universal laws and discrepancies. Front. Artif. Intell..

[24] Mangan NM, Brunton SL, Proctor JL, Kutz JN (2016). Inferring biological networks by sparse identification of nonlinear dynamics. IEEE Trans. Mol. Biol. Multi-Scale Commun..

[25] Qu Q, Sun J, Wright J (2016). Compressed sensing. IEEE Trans. Inf. Theory.

[26] Goyal P, Benner P (2022). Discovery of nonlinear dynamical systems using a Runge-Kutta inspired dictionary-based sparse regression approach. Proc. R. Soc. A Math. Phys. Eng. Sci..

[27] Leonhard, Methodus Inveniendi Lineas Curvas Maximi Minive Proprietate Gaudentes. *Bousquet, Lausanne & Geneva* (1744).

[28] Hills DJ, Grütter AM, Hudson JJ (2015). An algorithm for discovering Lagrangians automatically from data. PeerJ Comput. Sci..

[29] Ahmadi, M., Topcu, U. & Rowley, C. Control-oriented learning of Lagrangian and Hamiltonian systems. In *2018 Annual American Control Conference (ACC)* 520–525, (2018)

[30] Chu HK, Hayashibe M (2020). Discovering interpretable dynamics by sparsity promotion on energy and the Lagrangian. IEEE Robot. Autom. Lett..

[31] Kaiser E, Kutz JN, Brunton SL (2021). Data-driven discovery of Koopman eigenfunctions for control. Mach. Learn. Sci. Technol..

[32] Moore EH (1920). On the reciprocal of the general algebraic matrix. Bull. Am. Math. Soc..

[33] Kaheman K, Kutz JN, Brunton SL (2020). SINDy-PI: A robust algorithm for parallel implicit sparse identification of nonlinear dynamics. Proc. R. Soc. A Math. Phys. Eng. Sci..

[34] Fasel U, Kutz JN, Brunton BW, Brunton SL (2022). Ensemble-sindy: Robust sparse model discovery in the low-data, high-noise limit, with active learning and Control. Proc. R. Soc. A Math. Phys. Eng. Sci..

[35] Minguzzi E (2015). Rayleigh’s dissipation function at work. Eur. J. Phys..

[36] Hayashibe M, Shimoda S (2022). Synergetic synchronized oscillation by distributed neural integrators to induce dynamic equilibrium in energy dissipation systems. Sci. Rep..

[37] Friedman JH, Hastie T, Tibshirani R (2010). Regularization paths for generalized linear models via coordinate descent. J. Stat. Softw..

[38] Shor NZ (1998). Subgradient and $$\epsilon$$-Subgradient Methods.

[39] Beck A, Teboulle M (2009). A fast iterative shrinkage-thresholding algorithm for linear inverse problems. SIAM J. Img. Sci..

[40] Efron B, Hastie T, Johnstone I, Tibshirani R (2004). Least angle regression. Ann. Stat..

