# Supplementary material for: Sparse identification of Lagrangian for nonlinear dynamical systems via proximal gradient method
Source: Sci Rep. 2023 May 16;13:7919. doi: 10.1038/s41598-023-34931-0 (PMC10188520; doi:10.1038/s41598-023-34931-0)
Supplement: Supplementary file 3 — Supplementary Information 3. [file 41598_2023_34931_MOESM3_ESM.pdf]

Appendix: Derivation of Lagrangian

The Lagrangian is defined as $\mathcal{L} = T - V$, where T is the kinetic energy and V, is the potential energy. While the expression of the potential energy may vary depending on the type of conservative force acting on the system, the expression of the kinetic energy of a rigid body can be written as

$$T = \frac{1}{2}mv_{cm}^2 + \frac{1}{2}I\omega^2, \tag{1}$$

where m is the mass of the body, v_{cm} is the velocity of the center of mass, I is the moment inertia, and ω is the angular velocity. However, in the examples presented in this paper, we use a simplified pendulum where we can assume that the rod connecting the pendulum mass to its anchor is massless. Thus, we can assume that the pendulum only consists of point mass where the moment inertia of the rod is zero.

Single Pendulum

The coordinate of the single pendulum can be written as

$$x = L\sin\theta, \qquad y = -L\cos\theta. \tag{2}$$

By taking the time derivative of its position, we can get its velocity in horizontal and vertical directions respectively expressed as

$$\dot{x} = \dot{\theta}L\cos\theta, \qquad \dot{y} = \dot{\theta}L\sin\theta. \tag{3}$$

The kinetic energy and potential energy of the single pendulum can be written as

$$\begin{aligned} V &= mgy \\ &= -mgL\cos\theta, \end{aligned} \tag{4}$$

$$\begin{aligned} T &= \frac{1}{2}m(\dot{x}^2 + \dot{y}^2) \\ &= \frac{1}{2}m\left(\dot{\theta}^2L^2\cos^2\theta + \dot{\theta}^2L^2\sin^2\theta\right) \\ &= \frac{1}{2}mL^2\dot{\theta}^2. \end{aligned} \tag{5}$$

Using the parameter given in Fig. 2, the Lagrangian of this system can be written as

$$\mathcal{L} = 0.5\dot{\theta}^2 + 9.81\cos\theta. \tag{6}$$

Euler-Lagrange's equation of the single pendulum is

$$\frac{d}{dt}\frac{\partial\mathcal{L}}{\partial\dot{\theta}} - \frac{\partial\mathcal{L}}{\partial\theta} = 0. \tag{7}$$

By substituting eq. 6 to eq. 7, we can obtain

$$\ddot{\theta} + 9.81\sin\theta = 0, \tag{8}$$

and by solving for $\ddot{\theta}$, we get the equation of motion as follow

$$\ddot{\theta} = -9.81\sin\theta. \tag{9}$$

Cart Pendulum

The coordinate of the cart and the pendulum is respectively given by

$$x_c = x, \qquad y_c = 0, \tag{10}$$

$$x_p = x + L\sin\theta, \qquad y_p = -L\cos\theta, \tag{11}$$

and the time derivatives are given by

$$\dot{x_c} = \dot{x}, \qquad \dot{y_c} = 0, \tag{12}$$

$$\dot{x_p} = \dot{x} + \dot{\theta}L\cos\theta, \qquad \dot{y_p} = \dot{\theta}L\sin\theta. \tag{13}$$

The kinetic energy and the potential energy of the cart pendulum are expressed as

$$\begin{aligned} V &= m_c g y_c + m_p g y_p \\ &= -m_p g L\cos\theta, \end{aligned} \tag{14}$$

$$\begin{aligned} T &= \frac{1}{2}m_c(\dot{x_c}^2 + \dot{y_c}^2) + \frac{1}{2}m_p(\dot{x_p}^2 + \dot{y_p}^2) \\ &= \frac{1}{2}m_c\dot{x}^2 + \frac{1}{2}m_p\left(\dot{x}^2 + 2\dot{x}\dot{\theta}L\cos\theta + \dot{\theta}^2L^2\cos^2\theta + \dot{\theta}^2L^2\sin^2\theta\right) \\ &= \frac{1}{2}m_c\dot{x}^2 + \frac{1}{2}m_p\left(\dot{x}^2 + 2\dot{x}\dot{\theta}L\cos\theta + \dot{\theta}^2L^2\right) \\ &= \frac{1}{2}(m_c + m_p)\dot{x}^2 + m_p\dot{x}\dot{\theta}L\cos\theta + \frac{1}{2}m_p\dot{\theta}^2L^2. \end{aligned} \tag{15}$$

By plugging the parameter in Fig. 2, we obtain the Lagrangian as

$$\mathcal{L} = 0.75\dot{x}^2 + 0.5\dot{x}\dot{\theta}\cos\theta + 0.25\dot{\theta}^2 + 4.905\cos\theta. \tag{16}$$

Euler-Lagrange's equations of the cart pendulum with an external force on the cart are defined as

$$\frac{d}{dt}\frac{\partial\mathcal{L}}{\partial\dot{x}} - \frac{\partial\mathcal{L}}{\partial x} = F \tag{17}$$

and

$$\frac{d}{dt}\frac{\partial\mathcal{L}}{\partial\dot{\theta}} - \frac{\partial\mathcal{L}}{\partial\theta} = 0. \tag{18}$$

Inserting eq. 16 to eq. 17 and eq. 18, the generalized equation of motions are

$$1.5\ddot{x} + 0.5\ddot{\theta}\cos\theta - 0.5\dot{\theta}^2\sin\theta = F, \tag{19}$$

$$0.5\ddot{x}\cos\theta + 0.5\ddot{\theta} - 4.905\sin\theta = 0. \tag{20}$$

By solving for $\ddot{x}$ and $\ddot{\theta}$, the following equation of motions are obtained

$$\ddot{x} = \frac{F + 0.5\dot{\theta}^2\sin\theta - 4.905\cos\theta\sin\theta}{1 + \sin^2\theta}, \tag{21}$$

$$\ddot{\theta} = -\frac{F\cos\theta - 14.715\sin\theta + 0.5\dot{\theta}^2\cos\theta\sin\theta}{1 + \sin^2\theta}. \tag{22}$$

Double Pendulum

The coordinates of each pendulum respectively are given by

$$x_1 = L\sin\theta_1, \qquad y_1 = -L\cos\theta_1, \tag{23}$$

$$x_2 = L\sin\theta_1 + L\sin\theta_2, \qquad y_2 = -L\cos\theta 1 - L\cos\theta_2, \tag{24}$$

and the respective velocity is expressed as

$$\dot{x_1} = \dot\theta_1 L\cos\theta_1, \qquad \dot{y_1} = \dot\theta_1 L\sin\theta_1, \tag{25}$$

$$\dot{x_2} = L(\dot\theta_1\cos\theta_1 + \dot\theta_2\cos\theta_2), \qquad \dot{y_2} = L(\dot\theta_1\sin\theta_1 + \dot\theta_2\sin\theta_2). \tag{26}$$

The potential energy and the kinetic energy can be obtained as

$$\begin{aligned} V &= m_1 g y_1 + m_2 g y_2 \\ &= -gL[(m_1+m_2)\cos\theta_1 + m_2\cos\theta_2], \end{aligned} \tag{27}$$

$$\begin{aligned} T &= \frac{1}{2}m_1(x_1^2+y_1^2) + \frac{1}{2}m_2(x_2^2+y_2^2) \\ &= \frac{1}{2}L^2\left[m_1\left(\dot\theta_1{}^2\cos^2\theta_1 + \dot\theta_1{}^2\sin^2\theta_1\right)\right. \\ &\quad + m_2\left(\dot\theta_1{}^2\cos^2\theta_1 + 2\dot\theta_1\dot\theta_2\cos\theta_1\cos\theta_2 + \dot\theta_2{}^2\cos^2\theta_2\right) \\ &\quad \left.+ m_2\left(\dot\theta_1{}^2\sin^2\theta_1 + 2\dot\theta_1\dot\theta_2\sin\theta_1\sin\theta_2 + \dot\theta_2{}^2\sin^2\theta_2\right)\right] \\ &= \frac{1}{2}L^2\left[(m_1+m_2)\,\dot\theta_1^2 + 2m_2\dot\theta_1\dot\theta_2\sin\theta_1\sin\theta_2 + 2m_2\dot\theta_1\dot\theta_2\cos\theta_1\cos\theta_2 + m_2\dot\theta_2^2\right]. \end{aligned} \tag{28}$$

Substituting the parameter given in Fig. 2 yields the following Lagrangian

$$\mathcal{L} = \dot\theta_1^2 + \dot\theta_1\dot\theta_2\sin\theta_1\sin\theta_2 + \dot\theta_1\dot\theta_2\cos\theta_1\cos\theta_2 + 0.5\dot\theta_2^2 + 19.62\cos\theta_1 + 9.81\cos\theta_2. \tag{29}$$

Assuming there is a torque acting on each joint of the pendulum, the Euler-Lagrange equations of the double pendulum are given by

$$\frac{d}{dt}\frac{\partial\mathcal{L}}{\partial\dot\theta_1} - \frac{\partial\mathcal{L}}{\partial\theta_1} = \tau_1 - \tau_2, \tag{30}$$

$$\frac{d}{dt}\frac{\partial\mathcal{L}}{\partial\dot\theta_2} - \frac{\partial\mathcal{L}}{\partial\theta_2} = \tau_2. \tag{31}$$

Substituting eq. 29 to eq. 30 and eq. 31 yield the following equations

$$2\ddot\theta_1 + \ddot\theta_2\cos(\theta_2-\theta_1) - \dot\theta_2^2\sin(\theta_2-\theta_1) + 19.62\sin\theta_1 = \tau_1 - \tau_2, \tag{32}$$

$$\ddot{\theta}_2 + \ddot{\theta}_1 \cos(\theta_2 - \theta_1) + \dot{\theta}_1^2 \sin(\theta_2 - \theta_1) + 9.81 \sin\theta_2 = \tau_2. \tag{33}$$

The equations of motion for the double pendulum are then expressed as

$$\begin{aligned} \ddot{\theta}_1 = \Big[&\tau_1 - \tau_2(1 + \cos(\theta_2 - \theta_1)) + \dot{\theta}_1^2 \sin(\theta_2 - \theta_1)\cos(\theta_2 - \theta_1) \\ &+ \dot{\theta}_2^2 \sin(\theta_2 - \theta_1) + 9.81 \sin\theta_2 \cos(\theta_2 - \theta_1) - 19.62 \sin\theta_1\Big] / \left[2 - \cos^2(\theta_2 - \theta_1)\right], \end{aligned} \tag{34}$$

$$\ddot{\theta}_2 = \Big[\tau_2(2 + \cos(\theta_2 - \theta_1)) - \tau_1 \cos(\theta_2 - \theta_1) - \dot{\theta}_2^2 \sin(\theta_2 - \theta_1)\cos(\theta_2 - \theta_1) \tag{35}$$

$$- 2\dot{\theta}_1^2 \sin(\theta_2 - \theta_1) + 19.62 \sin\theta_1 \cos(\theta_2 - \theta_1) - 19.62 \sin\theta_2\Big] / \left[2 - \cos^2(\theta_2 - \theta_1)\right]. \tag{36}$$

Spherical Pendulum

Since a spherical pendulum is a higher-dimensional analog of a single pendulum. Thus it resides in a 3D cartesian coordinate. The coordinates of the pendulum are

$$\begin{aligned} x &= L \sin\theta \cos\phi, \\ y &= L \sin\theta \sin\phi, \\ z &= -L \cos\theta, \end{aligned} \tag{37}$$

and the respective time derivative is given by

$$\begin{aligned} \dot{x} &= L(\dot{\theta} \cos\theta \cos\phi - \dot{\phi} \sin\theta \sin\phi), \\ \dot{y} &= L(\dot{\theta} \cos\theta \sin\phi + \dot{\phi} \sin\theta \cos\phi), \\ \dot{z} &= L\dot{\theta} \sin\theta. \end{aligned} \tag{38}$$

The kinetic energy and the potential energy of the sphere pendulum are given by

$$\begin{aligned} V &= mgz \\ &= -mgL\cos\theta, \end{aligned} \tag{39}$$

$$\begin{aligned} T &= \frac{1}{2} m(\dot{x}^2 + \dot{y}^2 + \dot{z}^2) \\ &= \frac{1}{2} mL^2(\dot{\theta}^2 \cos^2\theta \cos^2\phi - 2\dot{\theta}\dot{\phi} \cos\theta \sin\theta \cos\phi \sin\phi + \dot{\phi}^2 \sin^2\theta \sin^2\phi \\ &\quad + \dot{\theta}^2 \cos^2\theta \sin^2\phi + 2\dot{\theta}\dot{\phi} \cos\theta \sin\theta \cos\phi \sin\phi + \dot{\phi}^2 \sin^2\theta \cos^2\phi + \dot{\theta}^2 \sin^2\theta) \\ &= \frac{1}{2} mL^2(\dot{\theta}^2 + \dot{\phi}^2 \sin^2\theta). \end{aligned} \tag{40}$$

By plugging in the parameter, we will get the Lagrangian

$$\mathcal{L} = 0.5\dot{\theta}^2 + 0.5\dot{\phi}^2 \sin^2\theta + 9.81 \cos\theta. \tag{41}$$

The Euler-Lagrange equations for the spherical pendulum with an external rotational torque in z-direction are defined as

$$\frac{d}{dt}\frac{\partial \mathcal{L}}{\partial \dot{\theta}} - \frac{\partial \mathcal{L}}{\partial \theta} = 0, \tag{42}$$

$$\frac{d}{dt}\frac{\partial \mathcal{L}}{\partial \dot{\phi}} - \frac{\partial \mathcal{L}}{\partial \phi} = \tau_{ext}. \tag{43}$$

Substituting eq. 41 to eq. 42 and eq. 43 yields

$$\ddot{\theta} + \dot{\phi}^2 \sin\theta\cos\theta - 9.81\sin\theta = 0, \tag{44}$$

$$\ddot{\phi}\sin^2\theta + 2\dot{\phi}\dot{\theta}\sin\theta\cos\theta = \tau_{ext}. \tag{45}$$

The equations of motion for the spherical pendulum are then expressed as

$$\ddot{\theta} = 9.81\sin\theta - \dot{\phi}^2\sin\theta\cos\theta, \tag{46}$$

$$\ddot{\phi} = \frac{\tau_{ext} - 2\dot{\phi}\dot{\theta}\sin\theta\cos\theta}{\sin^2\theta}. \tag{47}$$
